# Convergence Analysis of Iterative Deep Learning Algorithms for Fully Nonlinear BSPDEs in Non-Markovian Utility Maximization

**DOI:** 10.1007/s10915-026-03416-3

**Published:** 2026-07-28

**Authors:** Jingtang Ma, Haofei Wu, H. Harry Zheng

**Affiliations:** 1 School of Mathematics, Big Data Laboratory on Financial Security and Behavior (Laboratory of Philosophy and Social Sciences, Ministry of Education), Southwestern University of Finance and Economics, Chengdu, 611130 China; 2 School of Mathematics, Southwestern University of Finance and Economics, Chengdu, 611130 China; 3 Present Address: CAS AMSS - PolyU Joint Laboratory of Applied Mathematics, The Hong Kong Polytechnic University Shenzhen Research Institute, Shenzhen, 518057 China; 4 Department of Mathematics, Imperial College, London, SW7 2BZ UK

**Keywords:** Utility maximization, Non-Markovian model, Fully nonlinear BSPDE, Policy iteration, Deep learning, Error estimate, Convergence analysis, 60H35, 65C30, 93E20

## Abstract

In utility maximization with non-Markovian setting, the value function satisfies a stochastic Hamilton-Jacobi-Bellman (HJB) equation, a fully nonlinear backward stochastic partial differential equation (BSPDE). We propose iterative deep learning algorithms for such BSPDEs and analyze their convergence. We derive the error estimate of the time discretization scheme for BSPDEs, that of the policy iteration scheme for discretized BSPDEs, and that of the iterative deep learning scheme for discretized BSPDEs. We also test the algorithms and show their convergence and accuracy with numerical examples, including Markovian Heston volatility model and non-Markovian rough volatility model.

## Introduction

In this paper we will develop the iterative deep learning algorithm for the following type of fully nonlinear BSPDEs (also called a stochastic HJB equation, see Peng [21]):

1 $$\begin{aligned} -dv(t,x) = \mathop {\sup }\limits _{\pi } \{ \frac{1}{2} (\sigma _t \pi x)^2 D_{xx}v+ r_t x D_xv+ \sigma _t \pi x (\lambda _t D_xv + \rho D_xa)\} \,dt - a(t,x)\,dW_t, \end{aligned}$$

where  *W* is a standard Brownian motion, (v,a) a pair of progressively measurable random fields, $r_t$, $\sigma _t$ and $\lambda _t$ adapted processes, $D_xv$ the first order derivative of *v* with respect to *x* and evaluated at (*t*, *x*), other terms similarly defined, $\rho \in [-1,1]$ a constant correlation coefficient. BSPDE (1) arises in utility maximization under non-Markovian models. For Markovian models, the dynamic programming principle (DPP) leads to a fully nonlinear partial differential equation (PDE), called the HJB equation, for the value function (see Pham [22] and references therein). For non-Markovian models, the DPP fails to hold for the full filtration, making the HJB approach inapplicable. Peng [21] is the first to show that the value function *v*(*t*, *x*) satisfies a fully nonlinear BSPDE (1) (see details in Sect. 2) and proves the existence and uniqueness of solution pairs (*v*, *a*) when the coefficient of $D_{xx} v$ does not depend on control π. Qiu [24] uses random parabolic potentials to prove the existence and uniqueness of weak solutions to fully nonlinear BSPDEs in the presence of controlled leading coefficients. Three primary approaches in the literature for non-Markovian models are the Markovian approximation method [3], the path dependent PDE method [26, 27] and the rough PDE method [1]. These approaches rely on the special structure of Volterra stochastic integrals and are not applicable to general non-Markovian models. In this paper, we adopt the BSPDE approach of Peng [21], which is extendable to more general non-Markovian models.

We next give a brief literature review on the related work. E et al. [6] and Han et al. [10] are the first to propose the deep learning algorithms for backward stochastic differential equations (BSDEs) with applications to solving semi-linear parabolic PDEs. Beck et al. [5] extend the deep learning algorithms for second order BSDEs with applications to solving fully nonlinear PDEs. Huré et al. [14] introduce a novel deep learning algorithm called deep backward dynamic programming (DBDP) for BSDEs based on a classical backward resolution technique. These papers show the deep learning algorithms to have good numerical efficiency and accuracy but do not provide theoretical convergence and error analysis. Germain et al. [8] provide a rigorous theoretical foundation for the algorithm’s convergence. Han et al. [11] propose the deep backward regression-based method to solve high-dimensional quasilinear PDEs, which critically uses the conditional expectation representation of the pairs solutions of BSDEs and improves the deep learning BSDE method (Huré et al., 2020) by optimizing the local loss functions sequentially via backward time-stepping induction. The algorithms for BSDEs and BSPDEs are in general different. In fact, the neural network approximation for BSDEs is based on the link to the solution of semi-linear PDEs, but there is no such link for fully nonlinear BSPDEs that pose highly technical challenges in convergence analysis. For linear or quasilinear BSPDEs, Pardoux and Peng [23] show that the value functions of forward processes can be represented with BSDEs, which makes the deep learning BSDE approach in Huré et al. [14] applicable, with convergence analysis in the framework of Germain et al. [8]. For fully nonlinear BSPDEs, however, such BSDE representations are unavailable, rendering the techniques of Germain et al. [8] inapplicable. To the best knowledge of the authors, this is the first work in the literature on convergence analysis of the iterative deep neural network method for fully nonlinear BSPDEs.

Bayer et al. [4] address American option pricing under rough volatility models (a class of non-Markovian models) by formulating the problem as a reflected BSPDE, then approximating it via a sequence of penalized semi-linear BSPDEs, and finally solving these equations with the deep learning BSDE method of Huré et al. [14], supported with convergence results directly from Germain et al. [8]. Additionally, Li and Tang [16] propose a splitting algorithm for semi-linear BSPDEs, decomposing them into parameterized PDEs and BSDEs. Building on this idea, Zhang et al. [28] propose a deep learning method for semi-linear SPDEs by splitting them into parameterized PDEs and SDEs, and then transforming the PDEs into BSDEs that are solved with the deep learning algorithm of Huré et al. [14]. Li [17] introduces a local discontinuous Galerkin algorithm for linear BSPDEs and Sun et al. [25] utilize a finite element method in space and θ-scheme in time for semi-linear BSPDEs.

While all aforementioned papers focus on linear or semi-linear BSPDEs, our objective is to solve the fully nonlinear BSPDE (1), where significant theoretical and computational gaps remain. The key idea of our approach is to employ the Euler-Maruyama scheme for temporal discretization and the iterative deep neural network scheme for spatial approximation. The convergence analysis involves three crucial components: the error estimate for the Euler-Maruyama scheme (Theorem 3.1), that for the policy iteration scheme (Theorem 3.2), and that for the iterative deep learning scheme (Theorem 3.3). Combining these error estimates, we obtain the main convergence result with the geometric inequality (Theorem 3.4). The error estimate for the implicit Euler-Maruyama scheme is highly challenging, compared with those for linear and semi-linear BSPDEs (cf. Li and Tang [16], Zhang et al. [28], Li [17], Sun et al. [25]), due to the presence of fully nonlinear first- and second-order differential operators for random field solution pairs (*v*, *a*), which results in inconsistent metrics on both sides of the error equation, specifically, we need to use $L^2$ norm on the left side whereas $H^2$ norm on the right side to accommodate first- and second-order derivative terms. Under some appropriate assumptions, we get the error estimates on the right side with $L^2$ norm and derive the recursive error formula on the temporal grid, and then give the convergence order of the implicit Euler-Maruyama scheme by Gronwall’s inequality. Since the nonlinearity of discretized BSPDEs stems from the maximum of the Hamiltonian function, we propose a policy iteration scheme to get a sequence of linearized BSPDEs and show their solutions converge to those of fully nonlinear discretized BSPDEs. Finally, inspired by the deep learning BSDE method of Huré et al. [14], we design a deep learning BSPDE scheme for fully nonlinear discretized BSPDEs, coupled with policy iteration for linearized discretized BSPDEs, and give the error estimate of neural network approximation to the true solution (Theorem 3.4), which is markedly different from and much more difficult than the existing deep learning algorithms for linear or semi-linear BSPDEs. To the best knowledge of the authors, this is the first attempt in the literature in error and convergence analysis for the stochastic HJB equation, a fully nonlinear BSPDE, with deep learning and policy iteration schemes.

The remainder of the paper is organized as follows. In Sect. 2 we formulate fully nonlinear BSDPEs and construct iterative deep learning algorithms for their solutions. In Sect. 3 we state the convergence results of the algorithms, including the error estimate of the time discretization scheme for BSPDEs (Theorem 3.1), that of the policy iteration scheme for discretized BSPDEs (Theorem 3.2), and that of the iterative deep learning scheme for discretized BSPDEs (Theorem 3.3), and the main result of the paper, the error estimate of the iterative deep learning scheme for fully nonlinear BSPDEs (Theorem 3.4). In Sect. 4 we give two numerical examples, one Markovian Heston volatility model and one non-Markovian rough volatility model, and show the convergence and accuracy of the algorithm. In Sect. 5 we give the proofs of Theorems 3.1, 3.2, and 3.3. Section 6 concludes the paper. Appendix outlines the implementation of the iterative deep learning algorithm.

## BSPDE Formulation and Iterative Deep Learning Algorithms

Let $(\Omega ,\mathscr {F},(\mathscr {F}^*_t)_{t\ge 0},\mathbb {P})$ be a filtered complete probability space with $(\mathscr {F}^*_t)_{t\ge 0}$ being the natural filtration generated by two standard Brownian motions $W^0$ and *W* with correlation $\rho \in [-1,1]$ and $(\mathscr {F}_t)_{t\ge 0}$ being the natural filtration generated by *W*. Assume the market consists of a risk-free cash bond and a risky stock, whose price processes satisfy the following stochastic differential equations (SDEs), for $0\le t\le T$,

2 $$\begin{aligned} dB_t = r_t B_t\,dt,\quad dS_t =\mu _t S_t\,dt+\sigma _t S_t\,dW_t^0, \end{aligned}$$

where the interest rate $r_t$, stock return rate $\mu _t$ and volatility $\sigma _t$ are nonnegative $\mathscr {F}_t$-adapted processes, possibly non-Markovian. Let *X* be the wealth process of a self-financing portfolio comprising the bond *B* and the stock *S*. Then the wealth process *X* satisfies the following SDE, for $0\le t\le T$,

$$\begin{aligned} dX_t = (r_t+\sigma _t\pi _t\lambda _t)X_t\,dt +\sigma _t\pi _t X_t\,dW_t^0, \end{aligned}$$

where $\pi _t$ is a $\mathscr {F}^*_t$ progressively measurable and squared integrable control process that represents the proportion of wealth $X_t$ invested in risky asset $S_t$, and $\lambda _t:=(\mu _t-r_t)/\sigma _t$ is the market price of risk at time *t*. Using the orthogonal decomposition $W_t^0 = \rho W_t + \sqrt{1-\rho ^2} W_t^1$, where $W_t^1$ is a standard Brownian motion in $(\Omega ,\mathscr {F},(\mathscr {F}^*_t)_{t\ge 0},\mathbb {P})$, independent of $W_t$, we can rewrite the wealth process *X* as

$$ dX_t = (r_t+\sigma _t\pi _t\lambda _t)X_t\,dt+\rho \sigma _t\pi _t X_t \,dW_t + \sqrt{1-\rho ^2} \sigma _t\pi _t X_t \,dW_t^1. $$

Consider the following utility maximization problem

$$\begin{aligned} \mathop {\sup }\limits _{\pi } \mathbb {E}[U(X_T)], \end{aligned}$$

where $$U\in C^2(0,+\infty )$$ is an increasing and strictly concave function satisfying the Inada condition $U^{\prime }\left(0\right)=+\infty$, $U^{\prime }\left(+\infty \right)=0$ and the growth condition $$U(x)< C(1+x^p)$$ for x>0 with constant C>0 and 0<p<1. Define a $$\mathscr {F}_t$$-adapted random field value function, for $$0\le t\le T$$ and x>0,

3 $$\begin{aligned} v(t,x):= \mathop {\sup }\limits _{\pi } \mathbb {E}[U(X_T)|\mathscr {F}_t,\, X_t=x]. \end{aligned}$$

Peng [21] shows that *v* satisfies the following fully nonlinear BSPDE, for $0\le t\le T$ and x>0,

4 $$\begin{aligned} -dv(t,x) = \mathop {\sup }\limits _{\pi } G(t,x,v(t,x),a(t,x);\pi )\,dt - a(t,x)\,dW_t, \end{aligned}$$

with the terminal condition v(T,x)=U(x), where *v*(*t*, *x*) and *a*(*t*, *x*) are $\mathcal{F}_t$-adapted processes,

5 $$\begin{aligned} G(t,x,\phi ,\varphi ;\pi ):= \frac{1}{2} (\sigma _t \pi x)^2 D_{xx}\phi + r_t x D_x\phi + \sigma _t \pi x (\lambda _t D_x\phi + \rho D_x\varphi ). \end{aligned}$$

Define,

$$ \mathcal {P}(t,x,\phi ,\varphi ):=\mathop {\arg \max }\limits _{\pi } G(t,x,\phi ,\varphi ;\pi ) = -\frac{\lambda _t D_x\phi +\rho D_x\varphi }{\sigma _t x D_{xx}\phi }, $$

then the optimal control in (4) is given by

6 $$\begin{aligned} \pi ^{*}(t,x) = \mathcal {P}(t,x,v(t,x),a(t,x)) \end{aligned}$$

and

$$G(t,x,v(t,x),a(t,x);\pi ^*(t,x)) =\sup _\pi G(t,x,v(t,x),a(t,x);\pi ).$$

See Peng [21] for detailed derivation of BSPDE (4) and the corresponding verification theorem. A solution to BSPDE (4) is defined as a pair of $\mathscr {F}_t$-adapted random fields (*v*, *a*), see Ma et al. [18].

Now we propose the iterative deep learning algorithms for solving BSPDE (4). To this end, we truncate the wealth space (0,+∞) into a bounded domain $\Xi := (x_{\min },x_{\max })$ with $0<x_{\min }<x_{\max }<+\infty$ and supply the terminal and boundary conditions to (4):

7 $$\begin{aligned}  &   v(T,x) = U(x),\quad \hbox {for}\;\; x\in \Xi ,\end{aligned}$$

8 $$\begin{aligned}  &   v(t,x) = U(x),\quad \hbox {for}\;\; x\in \partial \Xi ,\quad t\in [0,T]. \end{aligned}$$

We present the construction of deep learning algorithms in four steps.

*First, we propose the implicit Euler-Maruyama scheme.* Let $t_n:= n\Delta t,\ n=0,1,\ldots ,N$ be a given uniform mesh on [0, *T*] where *N* is a positive integer and Δt=T/N. We discretize the backward process (4) in time interval $[t_{n},t_{n+1}]$ by Euler-Muruyama scheme as, for $x\in \Xi$ and n=N-1,…,1,0,

9 $$\begin{aligned} v_{n+1}(x) - v_n(x)= - \sup _{\pi } G(t_n,x,v_n(x),a_n(x);\pi )\Delta t + a_n(x)\Delta W_{t_n}, \end{aligned}$$

where $\Delta W_{t_n} = W_{t_{n+1}} - W_{t_{n}}$. The terminal condition is $v_{N}(x) = v(t_N,x)$ and the boundary condition is $v_n(x)|_{\partial \Xi } = v(t_n,x)|_{x \in \partial \Xi }$, which is consistent with the terminal and boundary conditions (7) - (8).

*Second, we incorporate the policy iteration.* We apply the policy iteration in (9) on $[t_n,t_{n+1}]$ to give, for k=0,1,…,

10 $$\begin{aligned}  &   v_{n+1}(x) - v_n^{(k+1)}(x) \\= &   -G(t_n,x,v^{(k+1)}_n(x),a^{(k+1)}_n(x);\pi _n^{(k)}) \Delta t + a_n^{(k+1)}(x) \Delta W_{t_n} \nonumber \end{aligned}$$

with boundary conditions $v_n^{(k)}(x)|_{\partial \Xi } = v_n(x)|_{\partial \Xi }$ for k=1,2,…, and initial conditions

$$ v_{n}^{(0)}(x)= v_{n+1}(x),\quad a_n^{(0)}=0, $$

where the iterative control $\pi _n^{(k)}$ is given by $\pi _n^{(k)}:=\pi _n^{(k)}(x)=\mathcal {P}(t_n,x,v_n^{(k)}(x),a_n^{(k)}(x))$.

*Third, we introduce the neural network approximation.* We construct a feedforward neural network with input dimension $d_0$, output dimension $d_1$, and L-1 (L≥2) hidden layers with each layer having $\bar{n}$ neurons. The neural network is a function from $\mathbb {R}^{d_0}$ to $\mathbb {R}^{d_1}$ defined by composition of simple functions as

$$ x \in \mathbb {R}^{d_0} \mapsto A_L \circ \varrho \circ A_{L-1} \circ \cdots \circ \varrho \circ A_1(x) \in \mathbb {R}^{d_1}, $$

where, $A_1:\mathbb {R}^{d_0} \mapsto \mathbb {R}^{\bar{n}}$, $A_l:\mathbb {R}^{\bar{n}} \mapsto \mathbb {R}^{\bar{n}}$ for l=2,3,…,L-1, and $A_L:\mathbb {R}^{\bar{n}} \mapsto \mathbb {R}^{d_1}$ are affine transformations defined by

$$ A_l(x) = \omega _l x + \beta _l, $$

where the matrix $\omega _l$ and the vector $\beta _l$ are called weight and bias for the *l*-th layer of the network, respectively, and ϱ is activation function which is applied componentwise on the outputs of $A_l$ for l=1,2,…,L-1. The parameters of the neural network are denoted by $\theta = (\omega _l, \beta _l)_{l=1}^L$. Given $d_0, d_1, L$, and $\bar{n}$, the total number of parameters in a network is $\bar{N}_{L,\bar{n}} = (d_0+1)\bar{n} + (\bar{n}+1)\bar{n}(L-2) + (\bar{n}+1)d_1$ and thus $\theta \in \mathbb {R}^{\bar{N}_{L,\bar{n}}}$. We denote the set of all possible parameters by $\Theta _{L,\bar{n}}$, and if there are no constraints on parameters, there exists $\Theta _{L,\bar{n}} = \mathbb {R}^{\bar{N}_{L,\bar{n}}}$. The set of all such neural networks with $\theta \in \Theta _{L,\bar{n}}$ is denoted by $\mathcal{N}\mathcal{N}_{d_0,d_1,L,\bar{n}}^{\varrho }(\Theta _{L,\bar{n}})$.

Based on (10), for fixed *n* and *k*, n=N-1,…,0, k=0,1,…, we construct the neural network approximation. Let

$$ (\widetilde{v}_n^{(k+1)}(x;\theta _n),\,\widetilde{a}_n^{(k+1)} (x;\eta _n))\in \mathcal{N}\mathcal{N}_{1+3(n+1),1,L,\bar{n}}^{\varrho }(\Theta _{L,\bar{n}}) \times \mathcal{N}\mathcal{N}_{1+3(n+1),1,L,\bar{n}}^{\varrho }(\Theta _{L,\bar{n}}), $$

where the paths of $r_{t_j},\,\mu _{t_j}$ and $\sigma _{t_j}$ for j=0,1,…,n are also input of neural networks besides *x*, which gives $d_0=1+3(n+1)$. Then minimize the loss function

11 $$\begin{aligned} (\theta _n^{(k+1)},\,\eta _n^{(k+1)}):= &   \mathop {\arg \min }\limits _{\theta _n,\,\eta _n} \mathbb {E}\Vert \widetilde{v}_{n+1}(x)- [\widetilde{v}_n^{(k+1)}(x;\theta _n) + \widetilde{a}_n^{(k+1)}(x;\eta _n) \Delta W_{t_n}\nonumber \\  &   \quad - G(t_n,x,\widetilde{v}_n^{(k+1)}(x;\theta _n), \widetilde{a}_n^{(k+1)}(x;\eta _n); \widetilde{\pi }_{t_n}^{(k)}(x)) \Delta t]\Vert _{L^2}^{2}\nonumber \\  &   \quad + \mathbb {E}[\widetilde{v}_n^{(k+1)}(x;\theta _n) - U(x)]^2|_{\partial \Xi }, \end{aligned}$$

and denote

$$ \widetilde{v}_n^{(k+1)}(x):= \widetilde{v}_n^{(k+1)}(x;\theta _n^{(k+1)}),\quad \widetilde{a}_n^{(k+1)}(x):= \widetilde{a}_n^{(k+1)}(x;\eta _n^{(k+1)}) $$

where $\Vert \cdot \Vert _{L^2}:=[\int _{\Xi }(\cdot )^2 \,dx]^{\frac{1}{2}}$ and $\widetilde{\pi }_{t_n}^{(k)} = \widetilde{\pi }_{t_n}^{(k)}(x) = \mathcal {P}(t_n,x,\widetilde{v}_{n}^{(k)}(x),\widetilde{a}_{n}^{(k)}(x))$, and $\widetilde{v}_{n+1}(x)$ is the iterative solution with optimal neural network on the pre-sequential time interval $[t_{n+1},t_{n+2}]$, and on $[t_{N-1},t_{N}]$ let $\widetilde{v}_{N}(x) = v_{N}(x)$ to make the terminal condition hold.

*Finally, we derive the complete algorithms.* Combining the time discretization, policy iteration, and neural network, we give the iterative deep learning algorithm for BSPDE (4), see Appendix A.

## Convergence Analysis

In this section, we prove the convergence of the iterative deep learning algorithm (Algorithm 1). The primary sources of errors come from time discretization, the policy iteration, and neural network approximation. We estimate each error source individually and then combine them to establish the convergence results. To this end, we first state two assumptions needed in convergence analysis.

### Assumption 3.1

For any $t,\,t_1,\,t_2 \in [0,T]$ and p≥1, there exist constants $C_p>0$ (depending on *p*) and ς>0 (independent of *p*) such that, for $\phi =r, \mu , \sigma$, ϕ satisfies

$$\begin{aligned}  &   \mathbb {E} |\phi _{t}|^p \le C_p, \quad \mathbb {E}|\phi _{t_1}-\phi _{t_2}|^p \le C_p |t_1-t_2|^{p\varsigma }. \end{aligned}$$

### Assumption 3.2

The random field solution pair (*v*, *a*) of BSPDE (4) and the optimal control $\pi ^*$ in (6) satisfy the following conditions, for $(t,x) \in [0,T] \times \Xi$,

(i) *v*(*t*, *x*) is $C^4$ in *x* with $D_{xx} v(t,x) <0$ and *a*(*t*, *x*) is $C^3$ in *x* with $a(\cdot ,x), D_x a(\cdot ,x)$ and $D_{xx}a(\cdot ,x)$ are square-integrable for t∈[0,T] almost everywhere on $\Xi \times \Omega$.
(ii) $\pi ^{*}(t,x)$ is $C^2$ in *x* and there exist two positive constants *c* and *C*, independent of *t*, *x*, such that
  $$ c<| \pi ^{*}(t,x) |<C \quad \hbox {and}\quad |D_x \pi ^{*}(t,x)| + |D_{xx} \pi ^{*}(t,x)| \le C. $$
(iii) For any p≥2, there exists a constant $C_p>0$, dependent of *p* but independent of *t*, such that
  $$ \mathbb {E} \Vert D_{x}v(t,\cdot ) \Vert _{L^2}^{p} + \mathbb {E} \Vert D_{xx}v(t,\cdot ) \Vert _{L^2}^{p} + \mathbb {E} \Vert D_{x}^{(3)}v(t,\cdot ) \Vert _{L^2}^{p}+ \mathbb {E} \Vert D_{x}^{(4)}v(t,\cdot ) \Vert _{L^2}^{p}\le C_p, $$
  where $D_x^{(k)}$ denotes the *k*th-order derivative in *x*, and $\mathbb {E} \Vert \cdot \Vert _{L^2}^{p}:= \mathbb {E}[\int _{\Xi }(\cdot )^2 \,dx]^{\frac{p}{2}}$.
(iv) For any p≥2, there exist two constants $C_p>0$ and C>0, independent of *t*, such that
  $$ \mathbb {E} \Vert a(t,\cdot ) \Vert _{L^p}^{p} + \mathbb {E} \Vert D_{x}a(t,\cdot ) \Vert _{L^p}^{p} + \mathbb {E} \Vert D_{xx}a(t,\cdot ) \Vert _{L^p}^{p} + \mathbb {E} \Vert D_{x}^{(3)}a(t,\cdot ) \Vert _{L^p}^{p}\le C_p, $$
  and
  $$ \mathbb {E}\{|a(t_1,x)-a(t_2,x)|^p + |D_xa(t_1,x)-D_xa(t_2,x)|^p\} \le C|t_1-t_2|^{p\varsigma }, $$
  for any $t_1,\, t_2\in [0,T]$ where ς>0 takes the same value as that in Assumption 3.1.

We first give the error estimate of the time discretization scheme, which is the error due to solutions of discretized BSPDE (9) replacing the true solution of BSPDE (4).

### Theorem 3.1

(Convergence of the time discretization scheme for BSPDE (4)) Let Assumptions 3.1 and 3.2 be satisfied. Then the convergence of the time discretization (9) is estimated as

$$\begin{aligned} \mathbb {E}\Vert v(t_n,x)-v_n(x)\Vert _{L^2}^2 \le C \Delta t^{\min \{1,2\varsigma \}} \end{aligned}$$

for n=N-1,…,1,0, where *C* is a generic positive constant, independent of step size Δt.

We next give the error estimate of policy iteration scheme, which is the error due to solutions of policy iteration algorithm (10) replacing the true solution of discretized BSPDE (9).

### Theorem 3.2

(Convergence of the policy iteration scheme for discretized BSPDE (9)) Let Assumptions 3.1 and 3.2 be satisfied. For sufficiently small time-step size Δt>0, the sequences $\{v_n^{(k)}(x)\}_{k\in \mathbb {N}}$ generated by the policy iteration scheme (10) satisfy

12 $$\begin{aligned} \mathbb {E}\Vert v_n^{(k)}(x) - v_n(x) \Vert _{L^2}^2 \le C(L')^k, \quad k=1, 2,\ldots \end{aligned}$$

for n=N-1,…,1,0, where $0<L^{\prime }<1,C>0$ are constants, independent of iteration step *k* and time size Δt, and $$v_n(x)$$ is the solution of discretized BSPDE (9).

Note that $v_{n+1}(x)$ in (9) is a given function. If it is replaced by some other given function $\widetilde{v}_{n+1}(x)$ (e.g., its neural network approximation) with $\hat{v}_n(x)$ the corresponding solution of (9), that is,

13 $$\begin{aligned} \widetilde{v}_{n+1}(x) - \hat{v}_n(x) = - G(t_n,x,\hat{v}_n(x),\hat{a}_n(x); \hat{\pi }_{n}^{*}(x)) \Delta t + \hat{a}_n(x) \Delta W_{t_n} \end{aligned}$$

with $\hat{\pi }_{n}^{*}:=\hat{\pi }_{n}^{*}(x) = \mathcal {P}(t_n,x,\hat{v}_n(x),\hat{a}_n(x))$, then sequences $\{\hat{v}_n^{(k)}(x)\}_{k\in \mathbb {N}}$ generated by the policy iteration scheme (10) would also have estimate (12), that is,

14 $$\begin{aligned} \mathbb {E}\Vert \hat{v}_n^{(k)}(x) - \hat{v}_n(x) \Vert _{L^2}^2 \le C(\hat{L})^k, \quad k=1, 2,\ldots \end{aligned}$$

for n=N-1,…,1,0 and some constants C>0 and $0<\hat{L}<1$. For fixed *n* and *k*, n=N-1,…,0 and k=1,2,…, denote by $\widetilde{v}_n^{(k)}(x)$ a deep neural network approximation to $\hat{v}_n^{(k)}(x)$ and $\widetilde{v}_n^{(k)}(x)$ is determined by the loss function (11) that is known to converge to 0 when the neural network size tends to infinite by the universal approximation theorem (see Hornik et al. [12, 13], also Bayer et al. [4] and Huré et al. [14]). Define, for φ=v and *a*,

15 $$\begin{aligned} \varepsilon _{n,\varphi }^{\mathcal {N},(k)} := \mathbb {E} \Vert \widetilde{\varphi }_n^{(k)}(x) - \hat{\varphi }_n^{(k)}(x) \Vert _{L^2}^2. \end{aligned}$$

We can give the error estimate of iterative deep learning scheme, which is the error due to deep neural network approximations, coupled with policy iteration, replacing the true solution of discretized BSPDE (9). We can establish the following results.

### Theorem 3.3

(Convergence of the iterative deep learning scheme for discretized BSPDE (9)) Let Assumptions 3.1 and 3.2 be satisfied. For sufficiently small time-step size Δt:=T/N, the output results $\widetilde{v}_n(x):=\widetilde{v}_n^{(K_n)}(x)$, generated by Algorithm 1, satisfy

16 $$\begin{aligned} \mathbb {E} \Vert \widetilde{v}_n(x)-v_n(x) \Vert _{L^2}^2 \le C [N^2 (\hat{L})^{K_n} + N^2 \varepsilon _{n,v}^{\mathcal {N},(K_n)}] \end{aligned}$$

for n=N-1,…,1,0, where $v_n(x)$ is the solution of the time discretization scheme (9), $K_n$ the total policy iteration number at time $t_n$, $\varepsilon _{n,v}^{\mathcal {N},(K_n)}$ the neural network approximation error defined by (15), $0<\hat{L}<1, C>0$ constants, independent of $K_n$ and *N*.

Combining Theorems 3.1 and  3.3, we have the main convergence theorem of this paper.

### Theorem 3.4

(Convergence of the iterative deep learning algorithm for the FBSPDEs) Let Assumptions 3.1 and 3.2 be satisfied. For sufficiently small time-step size Δt:=T/N, the output results $\widetilde{v}_n(x):=\widetilde{v}_n^{(K_n)}(x)$, generated by Algorithm 1, satisfy

17 $$\begin{aligned} \mathbb {E} \Vert \widetilde{v}_n(x)-v(t_n,x) \Vert _{L^2}^2 \le C [N^2 (\hat{L})^{K_n} + N^2 \varepsilon _{n,v}^{\mathcal {N},(K_n)} + N^{-\min \{1,2\varsigma \}} ], \end{aligned}$$

for n=N-1,…,1,0, where $v(t_n,x)$ is the solution of BSPDE (4) at time $t_n$, $K_n$ the total policy iteration number at time $t_n$, $\varepsilon _{n,v}^{\mathcal {N},(K_n)}$ the neural network approximation error defined by (15), $0<\hat{L}<1, C>0$ constants, independent of $K_n$ and *N*.

### Proof

Using the geometric inequality gives that

$$ \mathbb {E} \Vert \widetilde{v}_n(x)-v(t_n,x) \Vert _{L^2}^2 \le 2\mathbb {E} \Vert \widetilde{v}_n(x)-v_n(x) \Vert _{L^2}^2 + 2\mathbb {E} \Vert v_n(x)-v(t_n,x) \Vert _{L^2}^2, $$

then utilizing Theorem 3.1 with Δt=T/N and Theorem 3.3 completes the proof. □

### Remark 3.1

The error bound in Theorem 3.4 shows that one should first choose the number of time steps *N* sufficiently large, such that the third term is sufficiently small, then fix *N* and choose the iteration number $K_n$ and the size of the neural networks sufficiently large to make the algorithm converge. Note that once *N* is chosen, overly improving the accuracy of the neural network approximation does not noticeably reduce the final error of the numerical solution, nor does excessive policy iteration. In contrast, according to (17), as *N* increases (i.e., Δt=T/N decreases), both the number of iteration steps $K_n$ and the size of the neural network must be increased accordingly, which is necessary to reduce the errors arising from the policy iteration and the neural network approximation, thereby ensuring the convergence of the algorithm.

### Remark 3.2

The state variable *X* in this paper represents the wealth, a one-dimensional variable. A natural question to ask is if the proposed method can be extended to higher-dimensional state spaces, e.g., multi-assets or multi-factors. We believe there is a strong potential this can be achieved. The reason is that higher-dimensional state spaces do not affect the theoretical analysis concerning the one-dimensional time discretization or the policy iteration. The main challenges are likely to be computational: a significant increase in runtime, and the need to redesign the neural network architecture to enhance computational efficiency.

## Numerical Examples

In this section, we implement the iterative deep learning algorithms for the BSPDE (4) and verify the convergence results by the numerical experiments.

### Example 4.1

In this example, we consider a Markovian stochastic local volati-lity model with $\sigma _t=\sqrt{Y_t}$ and the process *Y* follows a stochastic volatility model:

$$ dY_t = f(Y_t)\,dt+g(Y_t)\,dW_t, $$

where *f* and *g* are two continuous functions satisfying certain conditions that guarantee $Y_t$ to be positive. Then the BSPDE (4) with replacing *v*(*t*, *x*) defined in (3) by

$$ V(t,x,Y_t):=\mathop {\sup }\limits _{\pi } \mathbb {E}[U(X_T)|\mathscr {F}_t,\, X_t=x] $$

has the solution

$$ a(t,x) = g(Y_t) D_yV(t,x,Y_t), $$

where V=V(t,x,y) satisfies the deterministic HJB equation:

18 $$\begin{aligned} -D_tV= &   \mathop {\sup }\limits _{\pi }\{\frac{1}{2}[\sigma (y) \pi x]^2 D_{xx}V + \sigma (y) \pi x [\lambda (y)D_x V + \rho g(y) D_{xy}V] \} \\  &   {}+ r x D_xV + \frac{1}{2} g^2(y) D_{yy}V + f(y) D_yV \nonumber \end{aligned}$$

with the terminal condition V(T,x,y)=U(x). The HJB equation can be solved using policy iteration methods, such as those in Ma and Ma [19]. However, for non-Markovian processes, such as the rough volatility model (19), the solution to the BSPDE (4) cannot be expressed in such forms. In particular, if we take

$$\begin{aligned} dY_t = \kappa (\zeta -Y_t)\,dt + \nu \sqrt{Y_t}\,dW_t, \end{aligned}$$

where $\kappa ,\, \zeta ,\, \nu$ are given positive constants and satisfy Feller conditions which ensure that $Y_t$ is positive for t∈[0,T]. $\mu _t$ in (2) is taken as

$$\begin{aligned} \mu _t = r + Y_t \end{aligned}$$

with *r* being a nonnegative constant. This setting makes Assumption 3.1 to be true with ς=0.5. Then the HJB (18) has the explicit solution (see Kraft [15]) with power-type utility function.

In this example, we solve $v(0,X_0)$ in the BSPDE (4) using Algorithm 1 and compare with the exact solution $V(0,X_0,Y_0)$ of HJB equation (18) for different initial wealths $X_0$, investment periods *T* and computational domains Ω to verify the convergence results. Values of model parameters are taken as:

$$ r = 0,\,S_0=100,\, Y_0=0.04,\, \rho =0,\, \kappa =2,\, \zeta =0.5,\,\nu =1, $$

which satisfy the required Feller conditions. The utility function is taken as $U(x) = 2\sqrt{x}$. Then exact solutions v(t,x),a(t,x) and the optimal control $\pi ^*(t,x)$ of the BSPDE (4) under this model are given by

$$\begin{aligned} v(t,x)= &   V(t,x,Y_t) = 2 \sqrt{x} e^{A(t) Y_t + B(t)},\\ a(t,x)= &   \sqrt{Y_t} D_y V(t,x,Y_t) = 2A(t) \sqrt{xY_t} e^{A(t) Y_t + B(t)},\\ \pi ^*(t,x)= &   2, \end{aligned}$$

where *A*, *B* are the solutions of the following Riccati equation

$$ -A'(t) = \frac{1}{2}A^2(t) - 2 A(t) + \frac{1}{2},\quad -B'(t) = A(t), $$

with terminal conditions A(T)=B(T)=0, see Kraft [15] for details. Combining the moment generating function of Cox-Ingersoll-Ross process $Y_t$, it can be verified that Assumption 3.2 holds.

Considering two investment periods T=0.1 and T=0.25, and corresponding numbers of temporal grids are set as $N=5,\,10,\,20,\,40$ and $N=10,\,20,\,40,\,80$, respectively. Computational domains are taken as $\Omega _1=[1,9]$ and $\Omega _2=[0.01,16]$, and corresponding numbers of spacial samples I=1000 for $\Omega _1$ and I=4000 for $\Omega _2$. Initial wealths are selected as $X_0=2,\,5,\,8$. The number of Monte Carlo paths is 10000. The upper bound of epochs is set as 2000. Set the error tolerance of policy iteration $\epsilon = 10^{-4}$. We adopt a double-hidden-layer feedforward neural network (i.e., L=3), where the number of neurons $\bar{n}$ in each hidden layer is set to half the total number of neurons in the input and output layers. The tanh function is chosen as the activation function to guarantee multiple differentiability. We select the Adam optimizer in PyTorch and use the full-batch training with adaptive learning rates between 0.01 and 0.05. The relative error between the computation value (CompV) and the benchmark value (BenchV) at initial wealth is calculated by

$$ \hbox {Error}:= |\frac{\widetilde{v}_0^{N}(X_0) - v(0,X_0)}{v(0,X_0)}|. $$

According to the result of Theorem 3.1, two different numbers of temporal steps $N_1,\,N_2$ and their corresponding relative errors $\hbox {Error}_1,\,\hbox {Error}_2$ satisfy

$$ \frac{\hbox {Error}_1}{\hbox {Error}_2} = (\frac{N_1}{N_2})^{-\min \{\frac{1}{2},\varsigma \}}. $$

Thus the convergence order of temporal discretization could be calculated by

$$ \hbox {Order}:= |\frac{\log {(\hbox {Error}_1/\hbox {Error}_2)}}{\log {(N_1/N_2)}}|. $$

**Fig. 1:**
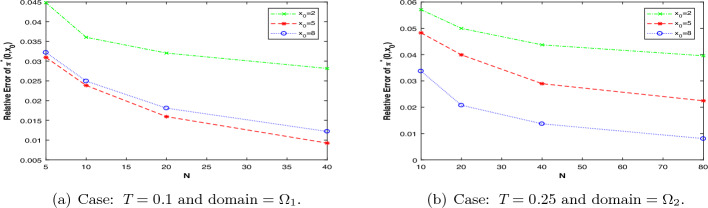
Convergence for the optimal controls for Example 4.1

**Fig. 2:**
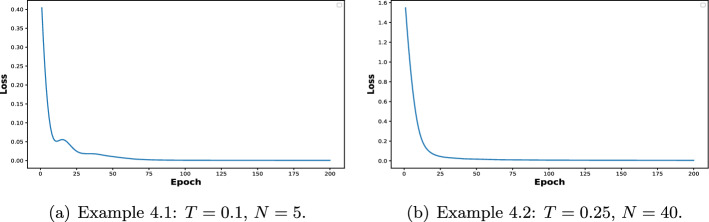
The averaged loss of training process

The average numbers of iteration policies (AvgIteN) are presented by

$$ \hbox {AvgIteN}:= \lceil \frac{1}{N} \sum _{n=0}^{N-1}K_n \rceil , $$

where *N* is the number of temporal steps and $K_n$ is the number of iteration policies at the temporal step $t_n$ for n=0,1,…,N-1, and $\lceil \cdot \rceil$ denotes the ceiling function. The benchmark values are calculated by the exact formula. The numerics in Table 1 show that the iterative deep learning algorithm is convergent with rates 1/2 in time and the policy iterations exhibit faster convergence, which are in agreement with Theorems 3.1 and 3.2. Moreover, it can be observed that for a given initial wealth $X_0$, the farther the boundaries of computational domain is placed away from $X_0$, the smaller the error is, which is due to the imposed boundary conditions are not that exact. Fig. 1 shows the relative error of computation for the optimal control decays as the number of time steps *N* increases, which verifies the convergence. Fig. 2 (a) shows that the loss averaged over all the policy iterations $k=1,2,\ldots ,K_n$ and all the time steps n=N-1,…,1,0 decreases to zero with the training epochs, which illustrates that the neural network is effective. Example 4.1 indicates that the method of BSPDEs and Algorithm 1 are applicable to the utility maximization on the incomplete market based on Markovian models.

From further numerical tests with increased numbers of layers *L* and those of neurons per layer $\bar{n}$, while keeping other parameters unchanged, we observe the essentially same numerical accuracy, which is consistent with the discussion in Remark 3.1, that is, “once *N* is chosen, overly improving the accuracy of the neural network approximation does not noticeably reduce the final error of the numerical solution, nor does excessive policy iteration."

**Table 1:** Convergence results of the iterative deep learning algorithm for Example 4.1

*T*	$X_0$	BenchV	*N*	CompV		Error		Order		AvgIteN	
				$\Omega _1$	$\Omega _2$	$\Omega _1$	$\Omega _2$	$\Omega _1$	$\Omega _2$	$\Omega _1$	$\Omega _2$
0.1	2	2.8402	5	2.8911	2.8834	1.792e−2	1.521e−2	-	-	21	23
			10	2.8747	2.8697	1.214e−2	1.039e−2	0.562	0.550	15	16
			20	2.8651	2.8615	8.761e−3	7.517e−3	0.471	0.467	12	12
			40	2.8582	2.8559	6.349e−3	5.518e−3	0.465	0.446	9	10
	5	4.4908	5	4.5487	4.5440	1.289e−2	1.185e−2	-	-	21	23
			10	4.5306	4.5276	8.872e−3	8.193e−3	0.539	0.532	15	16
			20	4.5201	4.5180	6.515e−3	6.061e−3	0.446	0.435	12	12
			40	4.5121	4.5108	4.739e−3	4.459e−3	0.459	0.443	9	10
	8	5.6804	5	5.7548	5.7360	1.310e−2	9.791e−3	-	-	21	23
			10	5.7312	5.7187	8.947e−3	6.737e−3	0.550	0.539	15	16
			20	5.7172	5.7083	6.477e−3	4.916e−3	0.466	0.455	12	12
			40	5.7069	5.7007	4.672e−3	3.574e−3	0.471	0.460	9	10
0.25	2	2.8778	10	2.9403	2.9312	2.172e−2	1.855e−2	-	-	24	25
			20	2.9213	2.9153	1.513e−2	1.302e−2	0.522	0.511	17	19
			40	2.9092	2.9047	1.092e−2	9.339e−3	0.470	0.479	13	14
			80	2.9017	2.8980	8.311e−3	7.018e−3	0.394	0.412	9	10
	5	4.5502	10	4.6159	4.6138	1.444e−2	1.398e−2	-	-	24	25
			20	4.5961	4.5949	1.008e−2	9.815e−3	0.519	0.510	17	19
			40	4.5833	4.5822	7.273e−3	7.028e−3	0.471	0.482	13	14
			80	4.5752	4.5747	5.495e−3	5.377e−3	0.404	0.386	9	10
	8	5.7555	10	5.8691	5.8110	1.974e−2	9.651e−3	-	-	24	25
			20	5.8346	5.7945	1.375e−2	6.782e−3	0.522	0.509	17	19
			40	5.8117	5.7837	9.772e−3	4.901e−3	0.493	0.469	13	14
			80	5.7986	5.7774	7.489e−3	3.799e−3	0.384	0.368	9	10

### Example 4.2

In this example, we consider a non-Markovian model with $\sigma _t=\sqrt{Y_t}$ and the process *Y* follows a rough volatility model (cf. El Euch and Rosenbaum [7]):

19 $$\begin{aligned} Y_t = Y_0 + \int _0^t \frac{(t-s)^{-\alpha }}{\Gamma (1-\alpha )} [\kappa (\zeta -Y_s)\,ds + \nu \sqrt{Y_s}\,dW_s], \end{aligned}$$

for $$0\le t \le T$$, $$Y_0>0$$ and $$\alpha \in (0,\frac{1}{2})$$.

We solve the BSPDE using Algorithm 1 and test the convergence by comparing with results in Han and Wong [9] at time t=0 for different initial wealths $X_0$, different investment periods *T* and different computational domains Ω. Values of some parameters are taken as $r = 0.02,\,\kappa =3,\,\rho =-0.6,\,\alpha =0.3$, and then $\varsigma = \frac{1}{2} - \alpha = 0.2$, and the values of other parameters are taken as the same as those in Example 4.1. Values of $\mu _t$ and $\sigma _t$ are obtained by solving the stochastic Volterra integral equation (19) using the fast algorithm in Ma and Wu [20]. The utility function is taken as $U(x) = 2\sqrt{x}$.

**Fig. 3:**
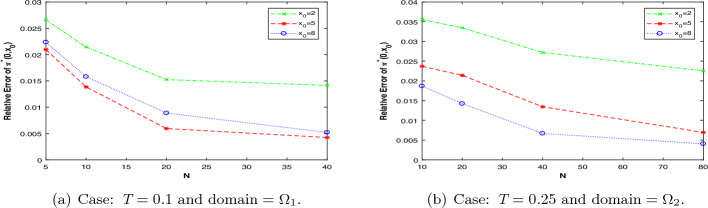
Convergence for the optimal controls for Example 4.2

The network, the error tolerance of policy iterations, initial wealth, investment periods, numbers of temporal grids and computational domains are set as same as Example 4.1. The benchmarks for values and controls are calculated by combining the results in Han and Wong [9] with Monte-Carlo methods. The average numbers of iteration policies are presented. Again the numerics in Table 2 show that the iterative deep learning algorithm is convergent and the orders are consistent with theoretical results. Fig. 3 confirms the convergence of the computation for optimal controls and Fig. 2 (b) shows that the neural network is effective. Results of Example 4.2 demonstrate that Algorithm 1 and the theoretical convergence results are also applicable to non-Markovian models.

**Table 2:** Convergence results of the iterative deep learning algorithm for Example 4.2

*T*	$X_0$	BenchV	*N*	CompV		Error		Order		AvgIteN	
				$\Omega _1$	$\Omega _2$	$\Omega _1$	$\Omega _2$	$\Omega _1$	$\Omega _2$	$\Omega _1$	$\Omega _2$
0.1	2	2.8496	5	2.8807	2.8739	1.091e−2	8.528e−3	-	-	24	25
			10	2.8744	2.8691	8.718e−3	6.847e−3	0.324	0.317	18	20
			20	2.8708	2.8663	7.428e−3	5.852e−3	0.231	0.227	13	14
			40	2.8681	2.8640	6.499e−3	5.063e−3	0.193	0.209	10	11
	5	4.5056	5	4.5357	4.5303	6.681e−3	5.482e−3	-	-	24	25
			10	4.5299	4.5253	5.395e−3	4.376e−3	0.308	0.325	18	20
			20	4.5264	4.5226	4.611e−3	3.765e−3	0.227	0.217	13	14
			40	4.5239	4.5205	4.072e−3	3.299e−3	0.179	0.191	10	11
	8	5.6991	5	5.7416	5.7219	7.457e−3	4.009e−3	-	-	24	25
			10	5.7334	5.7177	6.021e−3	3.259e−3	0.309	0.299	18	20
			20	5.7286	5.7150	5.176e−3	2.788e−3	0.218	0.225	13	14
			40	5.7253	5.7130	4.593e−3	2.446e−3	0.172	0.189	10	11
0.25	2	2.9036	10	2.9393	2.9279	1.230e−2	8.374e−3	-	-	25	27
			20	2.9321	2.9233	9.812e−3	6.790e−3	0.326	0.303	18	20
			40	2.9285	2.9205	8.573e−3	5.829e−3	0.195	0.220	15	15
			80	2.9261	2.9187	7.749e−3	5.192e−3	0.146	0.167	11	12
	5	4.5909	10	4.6293	4.6187	8.364e−3	6.055e−3	-	-	25	27
			20	4.6226	4.6138	6.908e−3	4.981e−3	0.276	0.282	18	20
			40	4.6192	4.6109	6.170e−3	4.366e−3	0.163	0.190	15	15
			80	4.6166	4.6091	5.602e−3	3.973e−3	0.139	0.160	11	12
	8	5.8071	10	5.8548	5.8332	8.214e−3	4.494e−3	-	-	25	27
			20	5.8460	5.8285	6.704e−3	3.681e−3	0.293	0.288	18	20
			40	5.8409	5.8259	5.822e−3	3.235e−3	0.204	0.186	15	15
			80	5.8371	5.8236	5.170e−3	2.842e−3	0.171	0.187	11	12

## Proofs of Theorems 3.1, 3.2, 3.3

### Proof of Theorem 3.1

To derive the error estimate for the time discretization scheme, we aim to establish a recurrence relation for the errors between consecutive time levels $t_n$ and $t_{n+1}$, and then apply Gronwall’s inequality. This requires consistency in the norms of the errors on both sides of the error equation (29). However, the left side is expressed in the $L^2$ norm, while the last four terms on the right side involve the $H^2$ norm. To resolve this discrepancy, we proceed as follows: first, we use integration by parts to handle the differential operators acting on the discretization errors, shown in (30)-(32); second, we use the regularity assumptions and Lemma 5.1 to estimate the truncation errors that include the last two terms in (37) and lead to (43); finally, we apply Gronwall’s inequality to (43) to give the convergence result for the time discretization scheme.

#### Lemma 5.1

Let Assumption 3.1 and Assumption 3.2 be satisfied. For any p≥2, we have

20 $$\begin{aligned} \mathop {\sup }\limits _{t\in [t_n,t_{n+1}]}\mathbb {E}\Vert v(t,x) - v(t_n,x)\Vert _{H^2}^p \le C\Delta t^{\frac{p}{2}}, \end{aligned}$$

and

21 $$\begin{aligned} \mathbb {E}\int _{t_n}^{t_{n+1}}\Vert a(t,x)-\bar{a}(t_n,x)\Vert _{H^1}^p\,dt \le C\Delta t^{1+p\varsigma }, \end{aligned}$$

where

22 $$\begin{aligned} \bar{a}(t_n,x) := \frac{1}{\Delta t}\mathbb {E}_n[\int _{t_n}^{t_{n+1}}a(t,x)\,dt], \end{aligned}$$

$\Vert \cdot \Vert _{H^1}:=\{ \int _{\Xi } \{ (\cdot )^2 + [D_x(\cdot )]^2\}\,dx\}^{\frac{1}{2}}$, $\Vert \cdot \Vert _{H^2}:= \{\int _{\Xi }\{(\cdot )^2 + [D_x(\cdot )]^2 + [D_{xx}(\cdot )]^2\}\,dx\}^{\frac{1}{2}}$, and $\mathbb {E}_n$ denotes the conditional expectation, given $\mathscr {F}_{t_n}$, and *C* denotes a generic positive constant that is independent of the time-step size Δt.

#### Proof

We first prove (20). To simplify notations, we denote, for all $t,t_n,x$, by

$$ v(t):=v(t,x),\ a(t):=a(t,x),\ \bar{a}(t_n):=\bar{a}(t_n,x), \ G(t):=G(t,x,v(t),a(t);\pi ^{*}(t)), $$

where $\pi ^{*}(t):=\mathop {\arg \sup }\limits _{\pi } G(t,x,v(t),a(t);\pi )$, see (6). (4) can be written as, for $t\in [t_n, t_{n+1}]$,

23 $$\begin{aligned} v(t)-v(t_n)=-[\int _{t_n}^{t}G(s)\,dt -a(s)d W_s]. \end{aligned}$$

According to the structure of *G* in (5) and Assumption 3.2, and combining Cauchy-Schwarz inequality and Hölder’s inequality, we can derive that for any t∈[0,T] and p≥2, there exist a positive constant *C* such that

24 $$\begin{aligned} \mathbb {E}\Vert G(t) \Vert _{H^2}^p \le C. \end{aligned}$$

Combining (23), for p>2, we can estimate that

$$\begin{aligned} \mathbb {E}\Vert [v(t)-v(t_n)]\Vert _{L^2}^p= &   \mathbb {E}[\int _{\Xi }(\int _{t_n}^{t}G(s)\,ds -a(s)\,dW_s)^{2}\,dx]^{\frac{p}{2}}\nonumber \\\le &   C\mathbb {E}[\int _{\Xi } (\int _{t_n}^{t}G(s)\,ds)^{2}dx]^{\frac{p}{2}} + C\mathbb {E} [\int _{\Xi } (\int _{t_n}^{t}a(s)\,d W_s)^{2}\,dx]^{\frac{p}{2}}\nonumber \\\le &   C\mathbb {E} [\int _{\Xi } \Delta t\int _{t_n}^{t}G^2(s)\,ds\,dx]^{\frac{p}{2}} + C\mathbb {E} [ \int _{\Xi }|\int _{t_n}^{t}a(s)\,d W_s|^{p}\,dx] \nonumber \\\le &   C\Delta t^{\frac{p}{2}}\mathbb {E}[\int _{t_n}^{t}\Vert G(s)\Vert _{L^2}^2\,ds]^{\frac{p}{2}} + C \int _{\Xi }\mathbb {E}[(\int _{t_n}^{t}a^2(s)\,ds )^{\frac{p}{2}}]\,dx\nonumber \\\le &   C\Delta t^{p-1}\int _{t_n}^{t}\mathbb {E} \Vert G(s)\Vert _{L^2}^p\,ds + C \Delta t^{\frac{p-2}{2}}\int _{t_n}^{t}\mathbb {E} \Vert a(s)\Vert _{L^p}^p\,ds\nonumber \\\le &   C\Delta t^{\frac{p}{2}}, \end{aligned}$$

where the first inequality is given by the geometric inequality, the second by Cauchy-Schwarz inequality $\int _{t_n}^t (\cdot )\,ds^2 \le \Delta t \int _{t_n}^t (\cdot )^2\,ds$ for $t\in [t_n,t_{n+1})$ and Hölder’s inequality $\int _{\Xi } (\cdot )^2 \,dx^{p/2} \le |\Xi |^{p/2-1} \int _{\Xi } (\cdot )^p \,dx$, the third by BDG inequality, the forth by Hölder’s inequality $\int _{t_n}^t (\cdot )^2\,ds^{p/2} \le \Delta t^{p/2-1} \int _{t_n}^t (\cdot )^p\,ds$ for $t\in [t_n,t_{n+1})$, and the last two by (24) and Assumption 3.2 (iv), also $\Delta t^{p}<\Delta t^{p/2}$ for Δt<1. We can similarly have

$$ \mathbb {E}\Vert D_x[v(t)-v(t_n)]\Vert _{L^2}^p + \mathbb {E}\Vert D_{xx}[v(t)-v(t_n)]\Vert _{L^2}^p \le C\Delta t^{p/2}, $$

which shows that (20) holds. Next we prove (21). Since

$$\begin{aligned} \Vert a(t)-\bar{a}(t_n)\Vert _{L^2}^p = (\int _{\Xi }\{a(t)-a(t_n) -\frac{1}{\Delta t}\mathbb {E}_n[\int _{t_n}^{t_{n+1}} a(t)-a(t_n)\,dt]\}^2\,dx)^{\frac{p}{2}}, \end{aligned}$$

we may repeatedly apply Jensen’s inequality, Cauchy-Schwarz inequality, Hölder’s inequality on the right side of the above equality and then use Assumption 3.2 (iv) to get

$$\begin{aligned} \mathbb {E}\int _{t_n}^{t_{n+1}}\Vert a(t)-\bar{a}(t_n)\Vert _{L^2}^p\,dt \le C \int _{\Xi }\mathbb {E}\int _{t_n}^{t_{n+1}} |a(t)-a(t_n)|^p\,dt\,dx \le C\Delta t^{1+p\varsigma }. \end{aligned}$$

We can similarly prove that

$$\mathbb {E}\int _{t_n}^{t_{n+1}}\Vert D_x [a(t)-\bar{a}(t_n)]\Vert _{L^2}^p\,dt \le C\Delta t^{1+p\varsigma }, $$

which shows that (21) holds. □

#### Proof of Theorem 3.1

To simplify notations, denote by $v_n:=v_n(x)$ and $a_n:=a_n(x)$. From (4) and (9), $v(t_n)$ and $v_n$ can be rewritten as

25 $$\begin{aligned} v(t_n)= &   v(t_{n+1}) + \int _{t_n}^{t_{n+1}} [G(t,x,v(t),a(t);\pi ^{*}(t))\,dt - a(t)\,dW_t],\end{aligned}$$

26 $$\begin{aligned} v_n= &   v_{n+1} + \int _{t_n}^{t_{n+1}} [G(t_n,x,v_n,a_n;\pi _n^{*})\,dt - a_n\,dW_t], \end{aligned}$$

where $\pi ^{*}(t):= \pi ^{*}(t,x) = \mathcal {P}(t,x,v(t),a(t))$ and $\pi _n^{*}:= \pi _n^{*}(x)= \mathcal {P}(t_n,x,v_n,a_n)$. To shorten notations, we denote, for all $n,t_n,x$,

$$ \Delta v_n(x):= v(t_n, x) - v_n(x),\quad \Delta a_n(x):= \bar{a}(t_n,x)-a_n(x), $$

we then have

27 $$\begin{aligned} \Vert \Delta v_n \Vert _{L^2}^2\le &   (\Delta v_{n+1}, \Delta v_n) + (\int _{t_n}^{t_{n+1}} a_n - a(t)\,dW_t,\Delta v_n) \nonumber \\+ &   (\int _{t_n}^{t_{n+1}} G(t,x,v(t),a(t);\xi _n^{*}(t))-G(t_n,x,v_n,a_n;\xi _n^{*}(t))\,dt, \Delta v_n), \end{aligned}$$

where $(\cdot ,\cdot ):= \int _{\Xi }\cdot \,\cdot \,dx$ is the inner product on Ξ, and for $t \in [t_n,t_{n+1})$,

$$\begin{aligned} \xi _n^{*}(t):= \left\{ \begin{array}{ll} \pi ^{*}(t),& \hbox {if} \;\, \Delta v_n(x)>0, \\ \pi _n^{*},&  \hbox {if} \;\, \Delta v_n(x)<0. \end{array} \right. \end{aligned}$$

Since $\pi _n^{*}$ is merely the temporal discretization of $\pi ^{*}(t)$, $\pi _n^{*}$ has the same regularity with respect to *x* as $\pi ^{*}(t)$ given in Assumptions 3.2 (ii). The spatial interval Ξ can be divided by intervals separated by zeros of $v(t_n)-v_n$ in *x* and, on each subinterval, $\xi _n^{*}(t)$ is $C^2$ and satisfies Assumptions 3.2 (ii). We can use integration by parts on each subinterval, then add them together to get the integral on the whole interval Ξ. Without loss of generality, we assume $\xi _n^{*}(t)$ is $C^2$ and has bounded derivatives on the whole interval Ξ.

We now estimate the third term in (27).

28 $$\begin{aligned}  &   \int _{t_n}^{t_{n+1}} G(t,x,v(t),a(t);\xi _n^{*}(t))-G(t_n,x,v_n,a_n;\xi _n^{*}(t))\,dt \nonumber \\= &   \int _{t_n}^{t_{n+1}} G(t,x,v(t),a(t);\xi _n^{*}(t))-G(t_n,x,v(t_n),\bar{a}(t_n);\xi _n^{*}(t))\,dt\nonumber \\  &   {}+ \int _{t_n}^{t_{n+1}} G(t_n,x,v(t_n),\bar{a}(t_n);\xi _n^{*}(t))-G(t_n,x,v_n,a_n;\xi _n^{*}(t))\,dt\nonumber \\= &   \int _{t_n}^{t_{n+1}} G(t,x,v(t),a(t);\xi _n^{*}(t))-G(t_n,x,v(t_n),\bar{a}(t_n);\xi _n^{*}(t))\,dt\nonumber \\  &   {}+ \int _{t_n}^{t_{n+1}} \frac{1}{2}[\sigma _{t_n} \xi _n^{*}(t) x]^2 D_{xx}\Delta v_n(x)\,dt\nonumber \\  &   {}+ \int _{t_n}^{t_{n+1}} [(\mu _{t_n}-r_{t_n}) \xi _n^{*}(t) x + r_{t_n} x] D_x\Delta v_n(x)\,dt\nonumber \\  &   {}+ \int _{t_n}^{t_{n+1}} \rho \sigma _{t_n} \xi _n^{*}(t) x D_x\Delta a_n(x)\,dt \nonumber \\=: &   \widetilde{\mathcal {T}}+\mathcal {T}_{1} +\mathcal {T}_{2} +\mathcal {T}_{3}, \end{aligned}$$

where $\bar{a}(t_n)$ is the average of *a*(*t*) over $[t_n,t_{n+1}]$, see (22). Incorporating (28) into (27) gives that

29 $$\begin{aligned} \Vert \Delta v_n\Vert _{L^2}^2\le &   (\Delta v_{n+1},\Delta v_n) +(\int _{t_n}^{t_{n+1}} a_n - a(t)\,dW_t,\Delta v_n) \nonumber \\  &   {}+ (\widetilde{\mathcal {T}},\Delta v_n) + (\mathcal {T}_1,\Delta v_n)+ (\mathcal {T}_2,\Delta v_n) + (\mathcal {T}_3,\Delta v_n). \end{aligned}$$

Now we estimate the RHS of (29). Denote by $\psi _1(x):= (\xi _n^{*}(t) x)^2$. Using integration by parts twice and $[v(t_n)-v_n]|_{\partial \Xi } = 0$, we have

30 $$\begin{aligned} (\mathcal {T}_1, \Delta v_n)= &   \int _{t_n}^{t_{n+1}} \int _{\Xi } \frac{1}{2}\sigma _{t_n}^2 \psi _1(x) \Delta v_n(x) D_{xx}\Delta v_n(x)\,dx\,dt\nonumber \\= &   \int _{t_n}^{t_{n+1}} \{\int _{\Xi } \frac{1}{4}\sigma _{t_n}^2 D_{xx} \psi _1(x) [\Delta v_n(x)]^2 \,dx - \int _{\Xi } \frac{1}{2} \sigma _{t_n}^2 \psi _1(x) [D_{x}\Delta v_n(x)]^2 \,dx \}\,dt. \end{aligned}$$

Denote by $\psi _2(x):= [(\mu _{t_n}-r_{t_n}) \xi _n^{*}(t) x + r_{t_n} x]$ and $\psi _3(x) = \rho \xi _n^{*}(t) x$. We can similarly get

31 $$\begin{aligned} (\mathcal {T}_2, \Delta v_n) = \int _{t_n}^{t_{n+1}} \int _{\Xi } -\frac{1}{2} D_{x} \psi _2(x) [\Delta v_n(x)]^2 \,dx\,dt \end{aligned}$$

and

32 $$\begin{aligned} (\mathcal {T}_3, \Delta v_n)= &   \int _{t_n}^{t_{n+1}} \int _{\Xi } - \sigma _{t_n}\{ D_{x} \psi _3(x) \Delta v_n(x) + \psi _3(x) D_x \Delta v_n(x)\} \Delta a_n(x)\,dx\,dt \nonumber \\\le &   \int _{t_n}^{t_{n+1}} \int _{\Xi } \frac{1}{2} [\sigma _{t_n} D_{x} \psi _3(x) \Delta v_n(x)]^2 \,dx \,dt \nonumber \\  &   {}- \int _{t_n}^{t_{n+1}} \int _{\Xi } \frac{1}{2}\sigma _{t_n}^2 \{[D_{x} \psi _3(x)]^2 + \psi _3(x) D_{xx} \psi _3(x)\} [\Delta v_n(x)]^2\,dx\,dt \nonumber \\  &   {}+ \int _{t_n}^{t_{n+1}} \int _{\Xi } \frac{1}{2} \sigma _{t_n}^2 \psi _3^2(x) [D_{x}\Delta v_n(x)]^2 \,dx \,dt + \frac{1}{2}\Delta t \int _{\Xi } [\Delta a_n(x)]^2\,dx. \end{aligned}$$

Combining (30), (31), and (32), using Cauchy-Schwarz inequality and Assumption 3.2, also noting $\psi _3^2(x) - \psi _1(x) = (\rho ^2-1) (\xi _n^{*}(t) x)^2 \le 0$ for any $\rho \in [-1,1]$, we have

33 $$\begin{aligned} \mathbb {E}(\mathcal {T}_1+\mathcal {T}_2+\mathcal {T}_3, \Delta v_n) \le C\Delta t \mathbb {E}\Vert \Delta v_n \Vert _{L^2}^2 + \frac{1}{2} \Delta t \mathbb {E}\Vert \Delta a_n \Vert _{L^2}^2. \end{aligned}$$

Now we estimate the second term of (33). To this end, multiplying both sides of (25) and (26) by $\Delta W_{t_n}$, taking conditional expectations with $\mathscr {F}_{t_n}$, and using Itô isometry, we have

$$\begin{aligned} a_n = \frac{1}{\Delta t} \mathbb {E}_n [v_{n+1} \Delta W_{t_n}], \end{aligned}$$

and

$$ \bar{a}(t_n) = \frac{1}{\Delta t} \mathbb {E}_n [v(t_{n+1}) \Delta W_{t_n}] + \frac{1}{\Delta t} \mathbb {E}_n [ \int _{t_n}^{t_{n+1}} G(t,v(t),a(t);\pi ^{*}(t)) \,dt \Delta W_{t_n}]. $$

Since

$$ \mathbb {E}_n \{\Delta W_{t_n} \mathbb {E}_n[\Delta v_{n+1}(x)]\}=0 $$

and

$$ \mathbb {E}_n \{\int _{t_n}^{t_{n+1}} G(t_n,v(t_n),a(t_n);\pi ^{*}(t_n)) \,dt \Delta W_{t_n}\}=0, $$

we have

34 $$\begin{aligned} \Delta t \Delta a_n(x) = \mathbb {E}_n \{\Delta W_{t_n}(\Delta v_{n+1}(x) - \mathbb {E}_n[\Delta v_{n+1}(x)])\} +\mathbb {E}_n \{ \int _{t_n}^{t_{n+1}} \Delta G(t,t_n) \,dt \Delta W_{t_n}\}, \end{aligned}$$

where $\Delta G(t,t_n):= G(t,x,v(t),a(t);\pi ^{*}(t))-G(t_n,x,v(t_n),a(t_n);\pi ^{*}(t_n))$. Taking expectations to the both sides of (34) and using Cauchy-Schwarz inequality and Young’s inequality: $(A+B)^2\le (1+\gamma \Delta t) A^2 + (1+\frac{1}{\gamma \Delta t}) B^2$ for some γ>0 to be chosen later and the law of iterated expectations, we derive that

35 $$\begin{aligned} \Delta t \mathbb {E}\Vert \Delta a_n \Vert _{L^2}^2\le &   \frac{1}{\Delta t} (1+\gamma \Delta t) \mathbb {E} \int _{\Xi }\mathbb {E}_n^2 \{\Delta W_{t_n}(\Delta v_{n+1}(x)-\mathbb {E}_n[\Delta v_{n+1}(x)])\}\,dx\nonumber \\  &   {}+ \frac{1}{\Delta t}(1+\frac{1}{\gamma \Delta t}) \mathbb {E} \int _{\Xi }\mathbb {E}_n^2 \{\int _{t_n}^{t_{n+1}} \Delta G(t,t_n) \,dt \Delta W_{t_n}\}\,dx\nonumber \\\le &   (1+\gamma \Delta t) \{\mathbb {E}\Vert \Delta v_{n+1}(x)\Vert _{L^2}^2 - \mathbb {E}\Vert \mathbb {E}_n[\Delta v_{n+1}(x)]\Vert _{L^2}^2\}\nonumber \\  &   {}+ (1+\frac{1}{\gamma \Delta t}) \mathbb {E} \int _{\Xi } \{\int _{t_n}^{t_{n+1}} \Delta G(t,t_n) \,dt\}^2 \,dx. \end{aligned}$$

Using the laws of iterated expectations, Cauchy-Schwarz inequality, Minkowski inequality, geometric inequality, and Young’s inequality, we have

36 $$\begin{aligned}  &   \mathbb {E} (\Delta v_{n+1}, \Delta v_n) + \mathbb {E} (\widetilde{\mathcal {T}}, \Delta v_n) \nonumber \\= &   \mathbb {E} (\mathbb {E}_n[\Delta v_{n+1}], \Delta v_n) + \mathbb {E} (\widetilde{\mathcal {T}}, \Delta v_n)\nonumber \\\le &   \frac{1}{2}\mathbb {E}\Vert \Delta v_n\Vert _{L^2}^2 + \frac{1}{2}(1+\gamma \Delta t) \mathbb {E}\Vert \mathbb {E}_n [\Delta v_{n+1}]\Vert _{L^2}^2 + \frac{1}{2}(1+\frac{1}{\gamma \Delta t}) \mathbb {E}\Vert \widetilde{\mathcal {T}} \Vert _{L^2}^2. \end{aligned}$$

Taking expectations to both sides of (29), noting that

$$\begin{aligned} \mathbb {E}(\int _{t_n}^{t_{n+1}} a_n - a(t)\,dW_t, \Delta v_n ) =\mathbb {E}(\mathbb {E}_n\int _{t_n}^{t_{n+1}} a_n - a(t)\,dW_t, \Delta v_n )=0, \end{aligned}$$

and combining (33), (35) and (36), we obtain that

37 $$\begin{aligned} \mathbb {E}\Vert \Delta v_n\Vert _{L^2}^2\le &   (\frac{1}{2}+C\Delta t )\mathbb {E}\Vert \Delta v_n\Vert _{L^2}^2 +\frac{1}{2}(1+\gamma \Delta t)\mathbb {E}\Vert \Delta v_{n+1}\Vert _{L^2}^2\nonumber \\  &   {}+ \frac{1}{2}(1+\frac{1}{\gamma \Delta t}) \mathbb {E} \int _{\Xi } \{\int _{t_n}^{t_{n+1}} \Delta G(t,t_n) \,dt\}^2 \,dx + \frac{1}{2} (1+\frac{1}{\gamma \Delta t})\mathbb {E}\Vert \widetilde{\mathcal {T}} \Vert _{L^2}^2. \end{aligned}$$

Next, we estimate the term $\mathbb {E} \Vert \widetilde{\mathcal {T}}\Vert _{L^2}^2$ in (37). Using Cauchy-Schwarz inequality and the geometric inequality gives that

38 $$\begin{aligned} \mathbb {E} \Vert \widetilde{\mathcal {T}}\Vert _{L^2}^2\le &   \mathbb {E} \int _{\Xi } \Delta t\int _{t_n}^{t_{n+1}} \{G(t,x,v(t),a(t);\xi _n^{*}(t))-G(t_n,x,v(t_n),\bar{a}(t_n);\xi _n^{*}(t))\}^2\,dt\,dx\nonumber \\\le &   2\Delta t \mathbb {E} \int _{\Xi } \int _{t_n}^{t_{n+1}} [G(t,x,v(t),a(t);\xi _n^{*}(t))-G(t_n,x,v(t),a(t);\xi _n^{*}(t))]^2\,dt\,dx\nonumber \\  &   {}+ 2\Delta t \mathbb {E} \int _{\Xi } \int _{t_n}^{t_{n+1}} [G(t_n,x,v(t)-v(t_n),a(t)-\bar{a}(t_n);\xi _n^{*}(t))]^2\,dt\,dx. \end{aligned}$$

Using the geometric inequality, Cauchy-Schwarz inequality and Hölder’s inequality, combining structures of *G* in (5), Assumptions 3.1 and 3.2, and Lemma 5.1, after some lengthy but straightforward estimation, we have

39 $$\begin{aligned} \mathbb {E} \int _{\Xi } \int _{t_n}^{t_{n+1}} [G(t,x,v(t),a(t);\xi _n^{*}(t))-G(t_n,x,v(t),a(t);\xi _n^{*}(t))]^2\,dt\,dx \le C\Delta t^{1+2\varsigma } \end{aligned}$$

and

40 $$\begin{aligned} \mathbb {E} \int _{\Xi } \int _{t_n}^{t_{n+1}} [G(t_n,x,v(t)-v(t_n),a(t)-\bar{a}(t_n);\xi _n^{*}(t))]^2\,dt\,dx \le C\Delta t^{\min \{2,1+2\varsigma \}}. \end{aligned}$$

Incorporating (39) and (40) into (38) gives that

41 $$\begin{aligned} \mathbb {E} \Vert \widetilde{\mathcal {T}}\Vert _{L^2}^2 \le C \Delta t^{\min \{3,2+2\varsigma \}}. \end{aligned}$$

Now we estimate the term $\mathbb {E} \int _{\Xi } \{\int _{t_n}^{t_{n+1}} \Delta G(t,t_n) \,dt\}^2 \,dx$ in (37). Combining Cauchy-Schwarz inequality, we have

$$\begin{aligned}  &   [\int _{t_n}^{t_{n+1}} \Delta G(t,t_n)\,dt]^2 \le \Delta t \int _{t_n}^{t_{n+1}} [\Delta G(t,t_n)]^2\,dt\\  &   \quad \le \Delta t \int _{t_n}^{t_{n+1}} [G(t,x,v(t),a(t);\widetilde{\xi }_n^{*}(t))-G(t_n,x,v(t_n),a(t_n);\widetilde{\xi }_n^{*}(t))]^2\,dt, \end{aligned}$$

where for $$t \in [t_n,t_{n+1})$$,

$$\begin{aligned} \widetilde{\xi }_n^{*}(t):= \left\{ \begin{array}{ll} \pi ^{*}(t),&  \hbox {if} \;\, \Delta G(t,t_n)>0, \\ \pi ^{*}(t_n),&  \hbox {if} \;\, \Delta G(t,t_n)<0. \end{array} \right. \end{aligned}$$

Similarly to the estimation of (38), using Assumptions 3.1 - 3.2 and Lemma 5.1, we derive that

42 $$\begin{aligned}  &   \mathbb {E} \int _{\Xi } [\int _{t_n}^{t_{n+1}} \Delta G(t,t_n) \,dt]^2 \,dx \nonumber \\\le &   \mathbb {E} \Delta t \int _{\Xi } \int _{t_n}^{t_{n+1}} [G(t,x,v(t),a(t);\widetilde{\xi }_n^{*}(t))-G(t_n,x,v(t_n),a(t_n);\widetilde{\xi }_n^{*}(t))]^2\,dt\,dx \nonumber \\\le &   C\Delta t^{\min \{3,2+2\varsigma \}}. \end{aligned}$$

Incorporating (41) and (42) into (37) and choosing γ=2C, we obtain that

43 $$\begin{aligned} \mathbb {E}\Vert \Delta v_n\Vert _{L^2}^2\le &   \frac{1+2C \Delta t}{1-2C\Delta t}\mathbb {E}\Vert \Delta v_{n+1}\Vert _{L^2}^2 + \frac{1+2C \Delta t}{1-2C\Delta t} \frac{C}{\Delta t} \Delta t^{\min \{3,2+2\varsigma \}} \nonumber \\\le &   \frac{1+2C \Delta t}{1-2C\Delta t}\mathbb {E}\Vert \Delta v_{n+1}\Vert _{L^2}^2 + C \Delta t^{\min \{2,1+2\varsigma \}}. \end{aligned}$$

Applying the discrete Gronwall’s inequality [2, Lemma 2.1] to inequality (43) gives that

$$\begin{aligned} \mathbb {E}\Vert v(t_n)-v_n\Vert _{L^2}^2 \le C\sum _{n=1}^{N} \Delta t^{\min \{2,1+2\varsigma \}} \le CT \Delta t^{\min \{1,2\varsigma \}}. \end{aligned}$$

The proof of this theorem is complete. □

### Proof of Theorem 3.2

#### Proof

To shorten notations, we denote, for all *n*, *x*, *k*,

$$\begin{aligned}  &   v_n^{(k)}:= v_n^{(k)}(x),\quad a_n^{(k)}:= a_n^{(k)}(x),\\  &   c_n^{(k)}:= \frac{1}{2} (\sigma _{t_n} \pi _n^{(k)} x)^2, \quad h_n^{(k)}:= (\mu _{t_n}-r_{t_n}) \pi _n^{(k)} x + r_{t_n} x,\\  &   \Delta v_n^{(k)}(x):= v_n^{(k)}(x) - v_n(x),\quad \Delta a_n^{(k)}(x):= a_n^{(k)}(x)-a_n(x). \end{aligned}$$

Then equations (9) and (10) are rewritten as

44 $$\begin{aligned}  &   v_{n+1}-v_n=-G(t_n,x,v_n,a_n;\pi _n^{*}) \Delta t + a_n \Delta W_{t_n},\end{aligned}$$

45 $$\begin{aligned}  &   v_{n+1}-v_n^{(k+1)}=-G(t_n,x,v_n^{(k+1)},a_n^{(k+1)};\pi _n^{(k)}) \Delta t + a_n^{(k+1)} \Delta W_{t_n}. \end{aligned}$$

Subtracting (44) by (45) gives that

46 $$\begin{aligned} 0= &   v_n^{(k+1)}-G(t_n,x,v^{(k+1)}_n,a_n^{(k+1)};\pi _n^{(k)}) \Delta t + a_n^{(k+1)} \Delta W_{t_n}\nonumber \\  &   {}- v_n+G(t_n,x,v_n,a_n;\pi _n^{*}) \Delta t - a_n \Delta W_{t_n}\nonumber \\= &   \Delta v_n^{(k+1)}(x) -G(t_n,x,\Delta v_n^{(k+1)}(x),\Delta a_n^{(k+1)}(x);\pi _n^{(k)}) \Delta t \nonumber \\  &   {}+ [G(t_n,x,v_n,a_n;\pi _n^{*}) - G(t_n,x,v_n,a_n;\pi _n^{(k)})] \Delta t + \Delta a_n^{(k+1)}(x) \Delta W_{t_n}. \end{aligned}$$

We first estimate the last term on the RHS of (46). Multiplying the equation (9) and (10) by $\Delta W_{t_n}$ and taking the expectations, we obtain that

$$\begin{aligned} a_n = a_n^{(k+1)} = \frac{1}{\Delta t} \mathbb {E}_n [v_{n+1} \Delta W_{t_n}],\quad \hbox {for}\;\, k=0,1,\ldots , \end{aligned}$$

which indicates that

47 $$\begin{aligned} \Delta a_n^{(k+1)}(x) = \Delta a_n^{(k)}(x) = 0. \end{aligned}$$

Furthermore, we tackle the third term on the RHS of (46). To this end, using Taylor expansions, we obtain that

48 $$\begin{aligned} G(t_n,x,v_n,a_n;\pi _n^{(k)})= &   G(t_n,x,v_n,a_n;\pi _n^{*}) + \frac{1}{2} (\sigma _{t_n}x)^2 D_{xx}v_n (\pi _n^{(k)} - \pi _n^{*})^2, \end{aligned}$$

49 $$\begin{aligned} G(t_n,x,v_n,a_n;\pi _n^{*})= &   G(t_n,x,v_n,a_n;\pi _n^{(k)}) + D_{\pi }G(t_n,x,v_n,a_n;\pi _n^{(k)}) (\pi _n^{*} - \pi _n^{(k)}) \nonumber \\  &   {}+ \frac{1}{2} (\sigma _{t_n}x)^2 D_{xx}v_n (\pi _n^{*} - \pi _n^{(k)})^2, \end{aligned}$$

where we have used

$$ D_{\pi }G(t_n,x,v_n,a_n;\pi _n^{*})=0 $$

and

$$ D_{\pi \pi }G(t_n,x,v_n,a_n;\pi )\equiv (\sigma _{t_n}x)^2D_{xx}v_n. $$

Adding (48) to (49) gives that

$$\begin{aligned} (\sigma _{t_n}x)^2 D_{xx}v_n (\pi _n^{*} - \pi _n^{(k)}) + D_{\pi }G(t_n,x,v_n,a_n;\pi _n^{(k)})= 0. \end{aligned}$$

Therefore we have

50 $$\begin{aligned} \pi _n^{(k)} - \pi _n^{*} = \frac{D_{\pi }G(t_n,x,v_n,a_n;\pi _n^{(k)})}{(\sigma _{t_n}x)^2 D_{xx}v_n} = \frac{D_{\pi }G(t_n,x,v_n-v_n^{(k)},a_n-a_n^{(k)};\pi _n^{(k)})}{(\sigma _{t_n}x)^2 D_{xx}v_n}, \end{aligned}$$

where we have used $D_{\pi }G(t_n,x,v_n^{(k)},a_n^{(k)};\pi _n^{(k)})=0$. On the other hand, the mean value theorem gives that

51 $$\begin{aligned} G(t_n,x,v_n,a_n;\pi _n^{(k)}) = G(t_n,x,v_n,a_n;\pi _n^{*}) + D_{\pi }G(t_n,x,v_n,a_n;\xi _{n}^{(k)}) (\pi _n^{(k)} - \pi _n^{*}), \end{aligned}$$

where $\xi _{n}^{(k)}$ is between $\pi _n^{*}$ and $\pi _n^{(k)}$. Plugging (50) into (51) and using the expression of $D_{\pi }G$ and the result in (47) give that

52 $$\begin{aligned} G(t_n,x,v_n,a_n;\pi _n^{(k)}) - G(t_n,x,v_n,a_n;\pi _n^{*}) = \widetilde{c}_n^{(k)} D_{xx}\Delta v_n^{(k)} + \widetilde{h}_n^{(k)} D_{x}\Delta v_n^{(k)}, \end{aligned}$$

where $\widetilde{c}_n^{(k)}:=-\pi _n^{(k)}[(\sigma _{t_n}x)^2 \xi _{n}^{(k)} + \sigma _{t_n}x\pi _n^{*}]$ and $\widetilde{h}_n^{(k)}:= - \lambda _{t_n} (\sigma _{t_n} x \xi _{n}^{(k)} + \pi _n^{*})$. Inserting (52) and (47) into (46) gives that

53 $$\begin{aligned}  &   \Delta v_n^{(k+1)}(x) -[c_n^{(k)} D_{xx}\Delta v_n^{(k+1)}(x) + h_n^{(k)} D_{x}\Delta v_n^{(k+1)}(x)] \Delta t \\= &   \Delta t [\widetilde{c}_n^{(k)} D_{xx}\Delta v_n^{(k)}(x) + \widetilde{h}_n^{(k)} D_{x}\Delta v_n^{(k)}(x)].\nonumber \end{aligned}$$

Taking the inner product with $v_n^{(k+1)}-v_n$ to the both sides of (53) gives that

$$\begin{aligned}  &   \Vert \Delta v_n^{(k+1)} \Vert _{L^2}^2 - \Delta t ( c_n^{(k)} D_{xx}\Delta v_n^{(k+1)} + h_n^{(k)} D_{x}\Delta v_n^{(k+1)}, \Delta v_n^{(k+1)} )\nonumber \\= &   \Delta t (\widetilde{c}_n^{(k)} D_{xx}\Delta v_n^{(k)} + \widetilde{h}_n^{(k)} D_{x}\Delta v_n^{(k)}, \Delta v_n^{(k)} ). \end{aligned}$$

Using integration by parts and Young’s inequality: $AB\le \frac{\xi }{2} A^2 + \frac{1}{2\xi } B^2$ for a positive constant ξ and combining the boundary conditions $[v_n^{(k+1)}-v_n]|_{\partial \Xi } = [v_n^{(k)} -v_n]|_{\partial \Xi } = 0$, we derive that

54 $$\begin{aligned}  &   \Vert \Delta v_n^{(k+1)} \Vert _{L^2}^2 + \Delta t \int _{\Xi } c_n^{(k)} [D_{x}\Delta v_n^{(k+1)}(x)]^2 \,dx \nonumber \\= &   \Delta t \int _{\Xi } \widetilde{c}_n^{(k)} D_{x} \Delta v_n^{(k)}(x) D_{x}\Delta v_n^{(k+1)}(x) \,dx \nonumber \\  &   {}+\Delta t \int _{\Xi } (\widetilde{h}_n^{(k)} - D_x\widetilde{c}_n^{(k)}) D_{x}\Delta v_n^{(k)}(x) \Delta v_n^{(k+1)}(x)\,dx\nonumber \\  &   {}+ \Delta t \int _{\Xi } (h_n^{(k)} - D_xc_n^{(k)}) \Delta v_n^{(k+1)}(x) D_x\Delta v_n^{(k+1)}(x) \,dx\nonumber \\\le &   \Delta t \frac{\xi _1}{2}\int _{\Xi } (\widetilde{c}_n^{(k)})^2 [D_{x}\Delta v_n^{(k)}(x)]^2 \,dx + \Delta t \frac{1}{2\xi _1}\int _{\Xi } [D_{x}\Delta v_n^{(k+1)}(x)]^2 \,dx\nonumber \\  &   {}+\Delta t \frac{\xi _2}{2}\int _{\Xi } (\widetilde{h}_n^{(k)} - D_x\widetilde{c}_n^{(k)})^2 [D_{x}\Delta v_n^{(k)}(x)]^2 \,dx + \Delta t \frac{1}{2\xi _2}\int _{\Xi } [\Delta v_n^{(k+1)}(x)]^2 \,dx\nonumber \\  &   {}+\frac{1}{2} \Delta t \int _{\Xi } (D_{xx}c_n^{(k)} - D_xh_n^{(k)}) [\Delta v_n^{(k+1)}(x)]^2 \,dx, \end{aligned}$$

where $\xi _1$ and $\xi _2$ are two positive constants to be determined later. Under assumptions on the boundedness of $\pi ^{*}(t)$, we can obtain that $\pi _n^{*}$ and $\pi _n^{(k)}$ are uniformly bounded, which then gives that

$$ \underline{c}\le c_n^{(k)},\quad (\widetilde{c}_n^{(k)})^2\le C_1,\quad (\widetilde{h}_n^{(k)} - D_x\widetilde{c}_n^{(k)})^2\le C_2,\quad |D_{xx}c_n^{(k)} - D_x h_n^{(k)}|\le C_3, $$

where $\underline{c},\, C_1,\, C_2,\, C_3$ are positive constants that are independent of iteration steps *k* and time-step size Δt. Then from (54) we get the following estimation

$$\begin{aligned}  &   \Vert \Delta v_n^{(k+1)} \Vert _{L^2}^2 + \underline{c} \Delta t |\Delta v_n^{(k+1)}|_{H^1}^2 \nonumber \\\le &   \Delta t \frac{\xi _1}{2} C_1 |\Delta v_n^{(k)}|_{H^1}^2 + \Delta t \frac{1}{2\xi _1}|\Delta v_n^{(k+1)}|_{H^1}^2\nonumber \\  &   {}+ \Delta t \frac{\xi _2}{2} C_2 |\Delta v_n^{(k)}|_{H^1}^2 + \Delta t \frac{1}{2\xi _2} \Vert \Delta v_n^{(k+1)}\Vert _{L^2}^2 + C_3 \Delta t \Vert \Delta v_n^{(k+1)}\Vert _{L^2}^2, \end{aligned}$$

which is rewritten equivalently as

55 $$\begin{aligned} (1-C_3\Delta t-\frac{1}{2\xi _2}\Delta t)\Vert \Delta v_n^{(k+1)} \Vert _{L^2}^2 + \Delta t (\underline{c}- \frac{1}{2\xi _1})|\Delta v_n^{(k+1)}|_{H^1}^2 \le \Delta t \frac{\xi _1 C_1 + \xi _2 C_2}{2} |\Delta v_n^{(k)}|_{H^1}^2, \end{aligned}$$

where $| \cdot |_{H^1}^2:= \int _{\Xi } [D_x(\cdot )]^2\,dx$. Now choose $\xi _1,\,\xi _2>0$ such that

$$ \underline{c}- \frac{1}{2\xi _1}> \frac{\xi _1 C_1 + \xi _2 C_2}{2} > 0, $$

then inequality (55) is equivalent to

56 $$\begin{aligned} |\Delta v_n^{(k+1)}|_{H^1}^2 + \frac{1-C_3\Delta t-\frac{1}{2\xi _2}\Delta t}{\Delta t (\underline{c}- \frac{1}{2\xi _1})}\Vert \Delta v_n^{(k+1)} \Vert _{L^2}^2 \le \frac{C_1\xi _1+C_2\xi _2}{2(\underline{c}- \frac{1}{2\xi _1})} |\Delta v_n^{(k)}|_{H^1}^2. \end{aligned}$$

Moreover for the above choice of $\xi _2$ and sufficiently small time-step size

$$\begin{aligned} \Delta t < \frac{2\xi _2(1-C_4)}{1+2\xi _2C_3}, \end{aligned}$$

with constant $0<C_4<1$, it can guarantee that

$$ 1-C_3\Delta t-\frac{1}{2\xi _2}\Delta t>C_4> 0. $$

Then from (56), we further estimate that

$$ |\Delta v_n^{(k+1)}|_{H^1}^2 + C'\Vert \Delta v_n^{(k+1)} \Vert _{L^2}^2 \le L' |\Delta v_n^{(k)}|_{H^1}^2 \le L' \{|\Delta v_n^{(k)}|_{H^1}^2 + C'\Vert \Delta v_n^{(k)} \Vert _{L^2}^2 \}, $$

where

$$ C':= \frac{C_4 (1+2\xi _2C_3)}{2\xi _2(1-C_4) (\underline{c}- \frac{1}{2\xi _1})},\quad L':= \frac{C_1\xi _1+C_2\xi _2}{2(\underline{c}- \frac{1}{2\xi _1})}. $$

Taking the expectations gives that

57 $$\begin{aligned} \mathbb {E}|\Delta v_n^{(k+1)}|_{H^1}^2 + C'\mathbb {E}\Vert \Delta v_n^{(k+1)} \Vert _{L^2}^2 \le L' \{\mathbb {E}|\Delta v_n^{(k)}|_{H^1}^2 + C'\mathbb {E}\Vert \Delta v_n^{(k)}\Vert _{L^2}^2 \}. \end{aligned}$$

Noting that $C^{\prime }>0$ and $0<L^{\prime }<1$. Inequality (57) recursively gives that

$$\begin{aligned} \mathbb {E}| \Delta v_n^{(k+1)}|_{H^1}^2 + C'\mathbb {E}\Vert \Delta v_n^{(k+1)} \Vert _{L^2}^2 \le (L')^{k+1} \{\mathbb {E}|v_{n+1} - v_n|_{H^1}^2 + C'\mathbb {E}\Vert v_{n+1} - v_n \Vert _{L^2}^2 \}. \end{aligned}$$

Therefore

$$ \mathbb {E}\Vert v_n^{(k+1)} - v_n \Vert _{L^2}^2 \le C(L')^{k+1}, $$

where *C* denotes a positive constant that is independent of *k* and Δt. The proof is thus complete. □

### Proof of Theorem 3.3

#### Proof

For convenience of writing, denote

$$\begin{aligned}  &   \hat{v}_n^{(k)}:= \hat{v}_n^{(k)}(x),\; \hat{a}_n^{(k)}:= \hat{a}_n^{(k)}(x),\; \widetilde{v}_n^{(k)}:= \widetilde{v}_n^{(k)}(x),\; \hat{v}_n:= \hat{v}_n(x), \; \hat{a}_n:= \hat{a}_n(x). \end{aligned}$$

Write

58 $$\begin{aligned} \widetilde{v}_n^{(k)}-v_n=[ \widetilde{v}_n^{(k)}-\hat{v}_n^{(k)}] + [ \hat{v}_n^{(k)}-\hat{v}_n] + [\hat{v}_n-v_n ]. \end{aligned}$$

The term $\widetilde{v}_n^{(k)}-\hat{v}_n^{(k)}$ is the error of neural network approximation, given by (15). The term $\hat{v}_n^{(k)}-\hat{v}_n$ is the error of policy iteration, given by (14). We only need to estimate the term $\hat{v}_n-v_n$. To this end, taking conditional expectation $\mathbb {E}_n$ to the auxiliary equation (13) and equation (9) gives that

$$\begin{aligned}  &   \hat{v}_{n}=\mathbb {E}_n[\widetilde{v}_{n+1}] + G(t_n,x,\hat{v}_{n},\hat{a}_{n};\hat{\pi }_{n}^{*}) \Delta t,\\  &   v_n = \mathbb {E}_n [v_{n+1}] + G(t_n,x,v_n,a_n;\pi _n^{*})\Delta t. \end{aligned}$$

Since $\pi _n^{*}= \mathop {\arg \sup }\limits _{\pi } G(t_n,x,v_n,a_n;\pi )$ and $\hat{\pi }_{n}^{*}=\mathop {\arg \sup }\limits _{\pi } G(t_n,x,\hat{v}_n,\hat{a}_n;\pi )$, we have

59 $$\begin{aligned}  &   \hat{v}_n - v_n \ge \mathbb {E}_n [\widetilde{v}_{n+1}-v_{n+1}]+ G(t_n,x,\hat{v}_n-v_n,\hat{a}_n-a_n;\pi _n^{*}) \Delta t, \end{aligned}$$

60 $$\begin{aligned}  &   \hat{v}_n - v_n \le \mathbb {E}_n [\widetilde{v}_{n+1}-v_{n+1}] + G(t_n,x,\hat{v}_n-v_n,\hat{a}_n-a_n;\hat{\pi }_{n}^{*}) \Delta t. \end{aligned}$$

Using (59) and (60) give that

61 $$\begin{aligned} \Vert \hat{v}_n - v_n \Vert _{L^2}^2\le &   (\mathbb {E}_n [\widetilde{v}_{n+1}-v_{n+1}], \hat{v}_n - v_n) \nonumber \\  &   {}+ \Delta t\int _{\Xi }G(t_n,x,\hat{v}_n-v_n,\hat{a}_n-a_n;\hat{\xi }_{n}^{*}) [\hat{v}_n - v_n]\,dx, \end{aligned}$$

where

$$\begin{aligned} \hat{\xi }_{n}^{*}= \left\{ \begin{array}{ll} \hat{\pi }_{n}^{*},&  \hbox {if} \;\, \hat{v}_n-v_n>0, \\ \pi _n^{*},&  \hbox {if} \;\, \hat{v}_n-v_n<0. \end{array} \right. \end{aligned}$$

Similarly to the estimation (33) in Theorem 3.1, taking expectations for (61), and using integrations by parts and boundary condition $[\hat{v}_n-v_n]|_{\partial \Xi } = 0$, and combining Cauchy-Schwarz inequality and the geometric inequality, we obtain that

62 $$\begin{aligned}  &   \mathbb {E} \Vert \hat{v}_n - v_n\Vert _{L^2}^2 \nonumber \\\le &   (\frac{1}{2} + C\Delta t) \mathbb {E} \Vert \hat{v}_n - v_n\Vert _{L^2}^2 + \frac{1}{2} \Delta t\mathbb {E} \Vert \hat{a}_n - a_n\Vert _{L^2}^2 + \frac{1}{2}\mathbb {E} \Vert \mathbb {E}_n [\widetilde{v}_{n+1}-v_{n+1}]\Vert _{L^2}^2 \nonumber \\  &   {}+\frac{1}{2} \Delta t (\rho ^2-1) \mathbb {E}\int _{\Xi } (\sigma _{t_n}\hat{\xi }_{n}^{*}x)^2 (D_x[\hat{v}_n - v_n])^2\,dx\nonumber \\\le &   (\frac{1}{2} + C\Delta t) \mathbb {E} \Vert \hat{v}_n - v_n\Vert _{L^2}^2 + \frac{1}{2} \Delta t\mathbb {E} \Vert \hat{a}_n - a_n\Vert _{L^2}^2 + \frac{1}{2}\mathbb {E} \Vert \mathbb {E}_n [\widetilde{v}_{n+1}-v_{n+1}]\Vert _{L^2}^2, \end{aligned}$$

as $\rho \in [-1,1]$. Moreover multiplying (13) and (9) by $\Delta W_{t_n}$ and then taking conditional expectation $\mathbb {E}_n$ give that

$$\begin{aligned} \hat{a}_n=\frac{1}{\Delta t}\mathbb {E}_n[\widetilde{v}_{n+1} \Delta W_{t_n}],\quad a_n=\frac{1}{\Delta t}\mathbb {E}_n[v_{n+1} \Delta W_{t_n}]. \end{aligned}$$

Furthermore, we obtain that

$$\begin{aligned} \Delta t(\hat{a}_n - a_n) = \mathbb {E}_n [\Delta W_{t_n} (\widetilde{v}_{n+1}-v_{n+1}) - \Delta W_{t_n} \mathbb {E}_n [\widetilde{v}_{n+1}-v_{n+1}]]. \end{aligned}$$

Further using Cauchy-Schwarz inequality and law of iterated expectation, it gives that

63 $$\begin{aligned} \Delta t\mathbb {E}\Vert \hat{a}_n - a_n\Vert _{L^2}^2 \le \mathbb {E}\Vert \widetilde{v}_{n+1}-v_{n+1}\Vert _{L^2}^2 - \mathbb {E} \Vert \mathbb {E}_n [\widetilde{v}_{n+1}-v_{n+1}]\Vert _{L^2}^2. \end{aligned}$$

Incorporating (63) into (62) gives that

64 $$\begin{aligned} \mathbb {E} \Vert \hat{v}_n - v_n\Vert _{L^2}^2 \le \frac{1}{1-C\Delta t}\mathbb {E} \Vert \widetilde{v}_{n+1}-v_{n+1}\Vert _{L^2}^2\le \frac{1+C\Delta t}{1-C\Delta t}\mathbb {E} \Vert \widetilde{v}_{n+1}-v_{n+1}\Vert _{L^2}^2. \end{aligned}$$

Inserting (64) into (58), and using Minkowski inequality and Theorem 3.2 give that

$$\begin{aligned} (\mathbb {E} \Vert \widetilde{v}_n^{(k)}-v_n\Vert _{L^2}^2)^{\frac{1}{2}}\le &   (\mathbb {E} \Vert \widetilde{v}_n^{(k)}-\hat{v}_n^{(k)}\Vert _{L^2}^2)^{\frac{1}{2}} + (\mathbb {E} \Vert \hat{v}_n^{(k)}-\hat{v}_n\Vert _{L^2}^2)^{\frac{1}{2}} + (\mathbb {E} \Vert \hat{v}_n-v_n\Vert _{L^2}^2)^{\frac{1}{2}}\\\le &   (\varepsilon _{n,v}^{\mathcal {N},(k)})^{\frac{1}{2}} +C(\hat{L})^{\frac{k}{2}} +\, \frac{1+C\Delta t}{1-C\Delta t} (\mathbb {E} \Vert \widetilde{v}_{n+1}-v_{n+1}\Vert _{L^2}^2)^{\frac{1}{2}}. \end{aligned}$$

Since $\widetilde{v}_n^{(K_n)}=\widetilde{v}_n$, choosing $k=K_n$ and using the discrete Gronwall’s inequality we get the estimation (16). The proof is complete. □

## Conclusions

In this paper, we have proposed iterative deep learning algorithms to solve the fully nonlinear BSPDEs arising from utility maximization under non-Markovian models. We establish the convergence of these algorithms, which is highly challenging due to the presence of fully nonlinear first- and second-order differential operators for random field solution pairs. By estimating the error contributions from time discretization, policy iteration, and neural network approximation, we establish the main convergence results. There remain many other open questions in this area, including prior estimates of number of policy iterations, size of neural networks, theoretical analysis of algorithmic stability, extension to non-Markovian optimal investment and stopping problems, etc., we leave these and other questions for future research.

## Data Availability

Data sharing not applicable - no new data generated, as the article describes entirely theoretical research.

## Iterative Deep Learning: Algorithm and Implementation

Let $\{\widetilde{v}_{n}(x),\widetilde{a}_{n}(x)\}$ be final output of the following iterative deep learning algorithm for BSPDE (4) whose exact solution at time $t_n$ is $\{v(t_n,x),a(t_n,x)\}$. Then the algorithm is given as follows.
